# Distribution Patterns of DNA N6-Methyladenosine Modification in Non-coding RNA Genes

**DOI:** 10.3389/fgene.2020.00268

**Published:** 2020-03-24

**Authors:** Yu Li, Xiao-Ming Zhang, Mei-Wei Luan, Jian-Feng Xing, Jianguo Chen, Shang-Qian Xie

**Affiliations:** ^1^Key Laboratory of Genetics and Germplasm Innovation of Tropical Special Forest Trees and Ornamental Plants (Ministry of Education), Hainan Key Laboratory for Biology of Tropical Ornamental Plant Germplasm, College of Forestry, Hainan University, Haikou, China; ^2^College of Grassland, Resources and Environment, Inner Mongolia Agricultural University, Huhhot, China; ^3^School of Life Sciences, Hubei University, Wuhan, China

**Keywords:** non-coding RNAs, model species, DNA methylation, gene expression, 6mA modification

## Abstract

N6-methyladenosine (6mA) DNA modification played an important role in epigenetic regulation of gene expression. And the aberrational expression of non-coding genes, as important regular elements of gene expression, was related to many diseases. However, the distribution and potential functions of 6mA modification in non-coding RNA (ncRNA) genes are still unknown. In this study, we analyzed the 6mA distribution of ncRNA genes and compared them with protein-coding genes in four species (*Arabidopsis thaliana*, *Caenorhabditis elegans*, *Drosophila melanogaster*, and *Homo sapiens*) using single-molecule real-time (SMRT) sequencing data. The results indicated that the consensus motifs of short nucleotides at 6mA location were highly conserved in four species, and the non-coding gene was less likely to be methylated compared with protein-coding gene. Especially, the 6mA-methylated lncRNA genes were expressed significant lower than genes without methylation in *A. thaliana* (*p* = 3.295e-4), *D. melanogaster* (*p* = 3.439e-11), and *H. sapiens* (*p* = 9.087e-3). The detection and distribution profiling of 6mA modification in ncRNA regions from four species reveal that 6mA modifications may have effects on their expression level.

## Introduction

DNA methylation, refers to the addition of a methyl group (CH3) to the DNA molecule, plays a critical role in epigenetic regulation of genes expression. Previous studies have paid more attention to 5-methylcytosine (5mC) in eukaryotes genomic DNA (gDNA) due to its abundance and significance (Zhang et al., 2006; Zilberman et al., 2007; Law and Jacobsen, 2010; Jones, 2012). In contrast, N6-methyladenosine (6mA) has been found at a significant level and commonly characterized in prokaryotes. Particularly, 6mA modifications in bacteria are involved in diverse biological processes such as DNA replication, DNA mismatch repair, host–pathogen interaction, and gene expression (Ratel et al., 2006; Wion and Casadesus, 2006). Recently, 6mA has been identified as a novel epigenetic mark in eukaryotes (Sun et al., 2015). And owing to the developed high-through sequencing technologies, 6mA modifications have been detected in diverse eukaryotes such as *Chlamydomonas* (Fu et al., 2015), *Arabidopsis thaliana* (Liang et al., 2018), *Caenorhabditis elegans* (Greer et al., 2015), *Drosophila melanogaster* (Zhang et al., 2015), *Mus musculus* (Koziol et al., 2016), and *Homo sapiens* (Xiao et al., 2018). These researches revealed that 6mA modifications did play important roles in regulating many biological processes in eukaryotes, such as the processes of embryonic development and tumorigenesis (Wu et al., 2016; Liang et al., 2018; Xiao et al., 2018).

Non-coding RNA (ncRNA) genes refer to genes that produce functional RNA sequences instead of translated proteins (Mattick and Makunin, 2006), which can be classified into ribosome RNA (rRNA), transfer RNA (tRNA), small nuclear RNA (snRNA), microRNA (miRNA), long non-coding RNA (lncRNA), and so on. In recent years, it has become increasingly apparent that ncRNAs are of significant functional importance in multicellular eukaryotes genome. And it has been demonstrated that ncRNAs involved diverse biological processes and the aberrational expression of non-coding genes were correlated with human diseases including cancer, lymphocytic leukemia, diabetes, band neurodegenerative diseases such as Alzheimer’s and Parkinson’s diseases (Dong et al., 2014; Chhabra, 2015; Wang and Chim, 2015; LaPierre and Stoffel, 2017). Recent studies have uncovered that DNA 5mC modification regulated the expression of both protein-coding genes and ncRNA genes (Borrello et al., 1992; Li et al., 2008; Chhabra, 2015). However, the 6mA distribution patterns in these ncRNA genes and regulatory relationships between 6mA and ncRNA genes remain unknown.

The single-molecule real-time (SMRT) sequencing technology, the third-generation sequencing platform, provides information regarding DNA modifications and identified 6mA and 4mC modifications at a single-nucleotide resolution and single-molecule level (Eid et al., 2009; van Dijk et al., 2018). The methylation signal was detected by the variations in inter-pulse duration (IPD) between two successive base incorporations during DNA synthesis (Flusberg et al., 2010; Clark et al., 2012; Fang et al., 2012; Schadt et al., 2013; Xiao et al., 2017). The developed SMRT sequencing technology allows genome-wide detection of 6mA at a high resolution. To date, the feature profiling of 6mA modification in some eukaryotes has been studied. However, the distribution pattern and potential function in ncRNA genes remain unknown. In this research, we identified the genome-wide 6mA modification sites in four species including *A. thaliana*, *C. elegans*, *D. melanogaster*, and *H. sapiens* by analyzing the SMRT sequencing datasets. Then we first decoded the distribution patterns of 6mA in ncRNA genes and compared them with protein-coding genes. The detection and distribution profiling of 6mA modification in ncRNA regions from four species reveal that 6mA modifications may have effects on their expression level.

## Materials and Methods

### Data Collection

We collected the paired long reads DNA and short-read RNA datasets from the same tissue of four eukaryotic species (*H. sapiens*, *D. melanogaster*, *C. elegans*, *A. thaliana*) from NCBI database (Supplementary Table S1). The raw DNA data sequenced by SMRT PacBio RSII were used to identify DNA methylated sites (Chin et al., 2016; Seo et al., 2016), and the short reads RNA datasets from the same tissue were used to explore the gene expression in *H. sapiens*, *D. melanogaster*, *C. elegans*, *A. thaliana* (Supplementary Table S1). The corresponding reference genomes and gene annotations were obtained from NCBI (Supplementary Table S1).

### Detection of DNA 6mA Modification

The PacBio SMRT analysis platform (version 2.3.0) was used to detect DNA 6mA modification^1^. The analysis pipeline was as follows: First, the raw SMRT sequencing datasets in h5 format downloaded from NCBI were filtered by using filter_plsh5.py with parameters: “-seed = 1 -minAccuracy = 0.75 -minLength = 50,” and the reads containing adapters, short reads (less than 50 nucleotide) or reads with a low quality region (less than 0.75) were removed. Second, the clean reads were aligned to the corresponding reference genome using pbalign with the parameters “-algorithmOptions = ‘-useQuality’ -algorithmOptions = ‘-minMatch 12 -bestn 10 -minPctIdentity 70.0’.” Then, the polymerase kinetics data were loaded by loadChemistry.py and loadPulses scripts with “-metrics DeletionQV, IPD, InsertionQV, PulseWidth, QualityValue, MergeQV, SubstitutionQV, DeletionTag.” Finally, the aligned datasets were sorted using cmph5tools, and 6mA sites were detected using ipdSummary.py script with “-methylFraction -identify 6mA -numWorkers 4.” Then we retained 6mA sites with more than 25-fold coverage for further analysis.

### Bioinformatics Analysis

For the profiling of 6mA in genomics features, we obtained the genome-wide methylation rate of adenine sites by calculating the mean of 6mA sites from all adenine sites. The genome-wide 6mA profiling across all chromosomes of *H. sapiens*, *D. melanogaster*, *C. elegans*, and *A. thaliana* were generated by using Circos (Krzywinski et al., 2009). For each 6mA modification site, we extracted 4 bp from the upstream and downstream sequences of the 6mA modification site as described in literature (Liang et al., 2018). The DREME was then used to predict conserved motifs in the flanking regions (Bailey, 2011). Besides, we used R 3.6.1 to perform the statistical analysis and figures drawing in this study.

### Non-coding RNA Genes Analysis

According to the annotation file (gff format) of the reference genome for each species, we divided genes into protein-coding genes, lncRNA, miRNA, snRNA, tRNA, and rRNA genes by using in-house shell scripts. The paired comparison analysis that compared the 6mA density (6mA/A) between ncRNA genes and protein-coding genes were tested by Student’s *t-*test. Furthermore, the lncRNA genes and protein-coding genes were divided into three and four groups, respectively, regarding to the gene length. Then, we analyzed the 6mA density of each gene length group and carried on *F*-test and Duncan multiple-range test after *F*-test was significant in any two groups.

### RNA-Seq Analysis

To explore the relationship between 6mA modification and gene expression in protein-coding genes and ncRNA, the clean RNA-seq reads were aligned to the reference genome using STAR (Dobin and Gingeras, 2015). The gene expression was calculated by Cufflinks (Trapnell et al., 2010) and the fragments per kilobase of transcript per million mapped reads (FPKM) was used to represent the gene expression abundance. The gene expression comparison between 6mA-methylated genes and -unmethylated genes was analyzed and tested by Student’s *t*-test.

## Results

### The Overview Characterization of 6mA in Four Species

**FIGURE 1:**
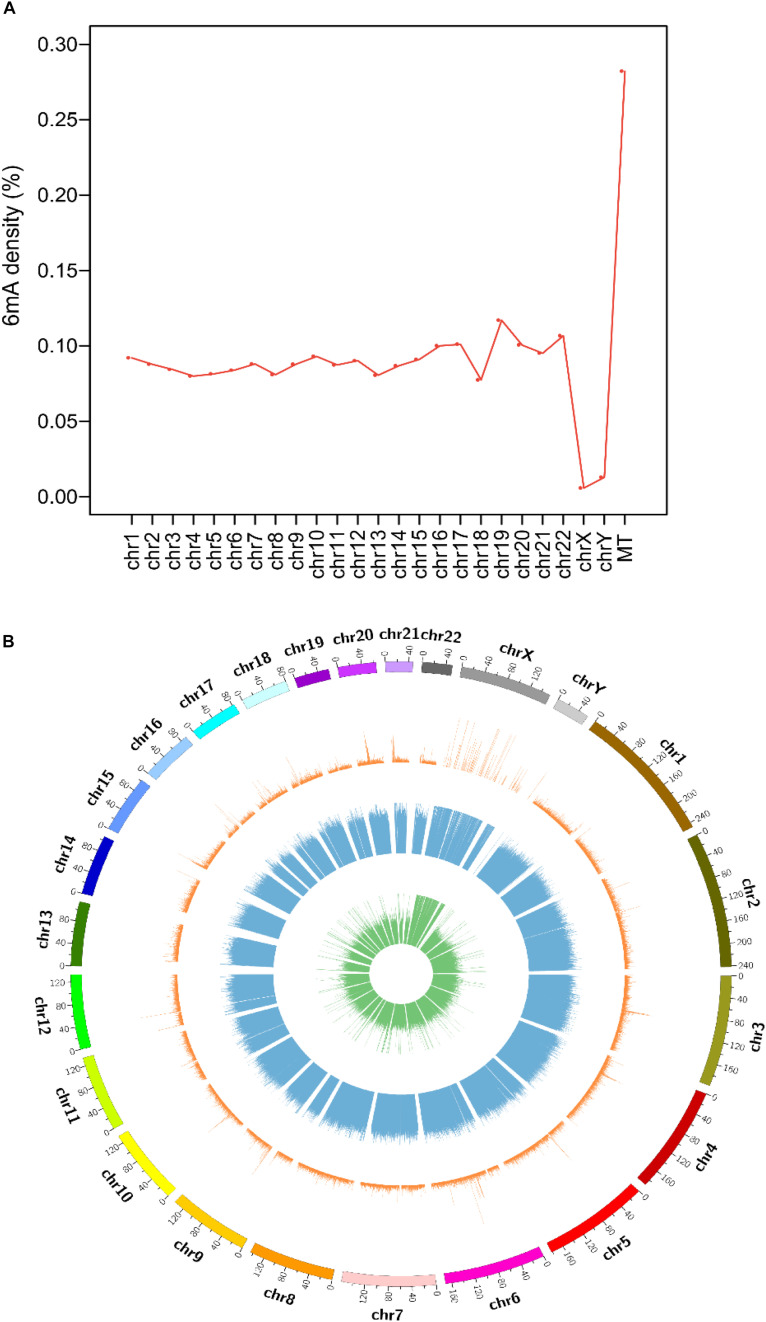
Distribution of N6-methyladenosine modification (6mA) in human genomic DNA. **(A)** Line diagram of 6mA modification density in human genomes. **(B)** Circos plot of 6mA in the human genome [green ring: lowly methylated (0–30%) 6mA; blue ring: moderately methylated (30–70%) 6mA; orange ring: highly methylated (70–100%) 6mA].

Collecting and analyzing the raw SMRT sequencing data of four species, we detected 75,630, 17,437, 18,226 and 1,439,519 6mA modification sites in *A. thaliana*, *C. elegans*, *D. melanogaster*, and *H. sapiens*, respectively (Table 1 and Supplementary Data Sheet S1). The 6mA density which refers to the number of adenines with 6mA methylation over all adenines (6mA/A) ranged from 0.023% in *D. melanogaster* to 0.099% in *A. thaliana* (Table 1). The 6mA density in *A. thaliana* genome was 0.099% and the chromosome 2 had the highest 6mA density 0.112% (Supplementary Figure S1A). In *C. elegans* genome, the density was 0.027% and chromosome 1 was with the high density 0.030% (Supplementary Figure S1B). In *D. melanogaster*, the gDNA density was 0.023% and the chromosome X had a high density of 0.029% (Supplementary Figure S1C). As for human, the 6mA density was 0.083% and chromosome 19 had the highest density 0.117%. In contrast, the chromosome X and Y with 6mA density 0.006 and 0.013%, respectively, which were remarkable lower than other chromosomes (Figure 1A). In all four species, the 6mA density in mitochondrial DNA was significantly higher than other chromosomes (Figure 1 and Supplementary Figure S1), and the 6mA density of chloroplast DNA was higher than mitochondrial DNA in *A. thaliana* (Supplementary Figure S1A).

**TABLE 1:** Statistical overview of 6mA modification in genomic DNA of four species.

**Species**	**Genome size (bp)**	**Total A number**	**6mA number**	**6mA ratio**
*A. thaliana*	119,668,627	76,401,859	75,630	0.099%
*C. elegans*	100,286,401	64,745,154	17,437	0.027%
*D. melanogaster*	137,567,484	79,393,495	18,226	0.023%
*H. sapiens*	3,088,286,401	1,737,096,659	1,439,519	0.083%

The distribution and modification level of 6mA sites in nuclear DNA in four species was profiled and showed in circos plot (Figure 1B and Supplementary Figure S2). According to the methylation level, 6mA sites were divided into three groups: low (0–30%, green ring), middle (30–70%, blue ring), and high (70–100%, orange ring). And we further profiled the 6mA density in each 50 kb bin in three 6mA methylation levels groups. Compared with high modification level, the low and middle modification levels were pervasively distributed in all chromosomes in four species. Particularly, the 6mA density in middle group was predominant which indicated the middle 6mA level was more variable across the genome in all species (Figure 1B and Supplementary Figure S2). Additionally, in human genome, the 6mA density of low and high modification level in the sexual chromosomes was different from the autosomal chromosomes (Figure 1B).

### Consensus Motifs for 6mA in Four Species

The consensus DNA sequence motifs of short nucleotides with a probable biological function were widespread around 6mA modification sites (Xiao et al., 2018). We extracted the upstream and downstream 4 bp sequences to investigate the enriched sequence motifs using DREME (Bailey, 2011) and further compared the motifs pattern in four species genomes.

**FIGURE 2:**
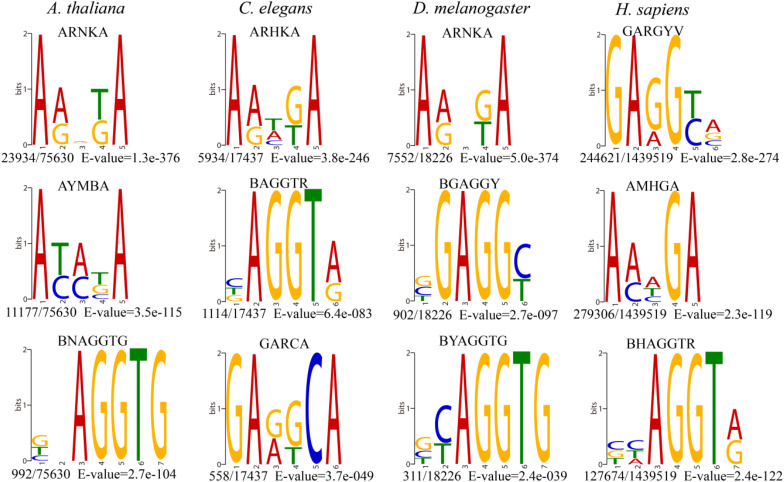
The top three enriched motifs of 6mA sites in four species. (The number of occurrences of each motif relative to the total number of 6mA-containing motifs and the corresponding E-value generated by DREME are shown under the sequence logo).

The most significantly enriched sequence motif AHNKA was identified in *A. thaliana* and *D. melanogaster* (Figure 2), which was also highly similar to the most enriched motif ARHKA in *C. elegans* and the second enriched motif AMHGA in *H. sapiens* (Figure 2). These motifs all contained two conserved adenines at both ends (Figure 2). In addition, the AGGT motif presented among the top three enriched motifs in four species shared the consensus core AGG with the motif sequence GAGG and AGGC (Figure 2 and Supplementary Figure S3). Above results illustrated that the 6mA modification in multicellular eukaryotes shared highly conserved sequence of short nucleotides, and further verified the reliability of the consensus motifs of 6mA sites.

### The Distribution Pattern of 6mA in Non-coding RNA Genes

**FIGURE 3:**
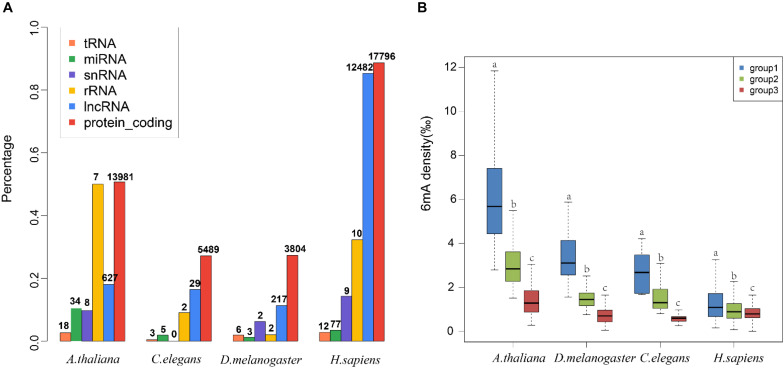
Distribution pattern of 6mA modification in non-coding genes in four species. **(A)** The percentages of genes containing 6mA modification in different gene categories were shown in histogram. The numbers of 6mA-methylated genes in different categories were shown above the histogram. **(B)** The 6mA density of different lncRNA gene length groups was shown in boxplot. The different letters shown above the box meant the significant difference in Duncan multiple-range test.

To decode the 6mA distribution pattern of ncRNA genes, we compared the ncRNA genes with protein-coding genes. Interestingly, the percentage of 6mA-methylated ncRNA genes was commonly smaller than protein-coding genes in four species (*p* < 2.2e-16) (Figure 3A and Supplementary Data Sheet S2), which may be correlated with the lower gene expression in ncRNA genes. However, the 6mA density in tRNA, miRNA, and snRNA gene was significantly higher than protein-coding genes in four species (Supplementary Figure S4A and Supplementary Data Sheet S3). For lncRNA genes, the 6mA density was significantly higher than protein-coding genes except for human, and the density in rRNA in *C. elegans* and *H. sapiens* was different from protein-coding genes (Supplementary Figure S3A and Supplementary Data Sheet S3). These results suggested that the 6mA modification could have specific function across ncRNA and protein-coding genes.

We further detailed the 6mA modification distribution in lncRNA and protein-coding genes. The lncRNA genes were classified into three groups regarding to the gene length (Supplementary Table S2). To ensure similar amounts of genes in each group, three groups were divided by length <500, 500–1000, and >1000 in *A. thaliana*, respectively. In *C. elegans* and *D. melanogaster*, group 1, 2, and 3 represented gene length < 1000, 1000–2000, >2000, respectively. In *H. sapiens*, group 1, 2, and 3 were classified with length < 10,000, 10,000–20,000, >20,000, respectively. The difference of 6mA density among different gene length groups was significant (Figure 3B and Supplementary Data Sheet S3). Meanwhile, we classified the protein-coding genes into four groups (Supplementary Table S2 and Supplementary Figure S4B). The results indicated that the protein-coding genes were consistent with the lncRNA genes that the short genes tended to contain higher 6mA density in four species (Figure 3B and Supplementary Figure S4B).

### Correlation Between 6mA Methylation and Gene Expression in lncRNA

**FIGURE 4:**
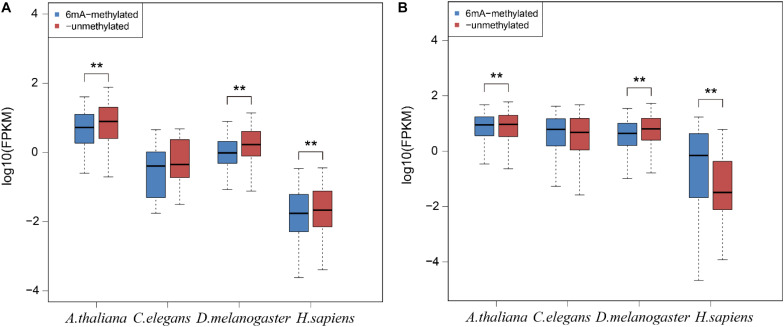
The correlation between 6mA modification and gene expression in lncRNA and protein-coding genes. **(A)** lncRNA genes. **(B)** Protein-coding genes. The *P*-value was calculated by Student’s *t-*test. **means that there was a significant diffrence between two groups.

To examine the relationship between 6mA modification and gene expression in lncRNA, we categorized all lncRNA and protein-coding genes into two groups: 6mA-methylated genes and non-6mA methylated genes (Supplementary Data Sheet S4). FPKM values of genes were calculated and compared between two categories. The 6mA-methylated lncRNA genes expressed lower than -unmethylated genes in all four species (Figure 4A). The differences in *A. thaliana* (*p* = 3.295e-4), *D. melanogaster* (*p* = 3.439e-11), and *H. sapiens* (*p* = 9.087e-3) were significant (Figure 4A). For protein-coding genes, the expression of 6mA-methylated protein-coding genes was significantly higher than -unmethylated genes (*p* < 2.2e-16) in *H. sapiens* (Figure 4B), which was consistent with previous study (Xiao et al., 2018). However, the opposite trend was observed in *A. thaliana* (*p* = 6.074e-15) and *D. melanogaster* (*p* < 2.2e-16) (Figure 4B) that 6mA-metyalted genes expressed significantly lower than -unmethylated genes, which was contrast to studies (Greer et al., 2015; Liang et al., 2018). And there was no significant difference between 6mA-methylated and unmethylated protein-coding genes which was the same as lncRNA genes in *C. elegans* (Figure 4). These results further indicated that 6mA modification might have specific function across lncRNA and protein-coding genes and illustrated that the correlation between 6mA and gene expression in various organisms and different individuals in the same organism was dynamical.

## Discussion

DNA 6mA modification had been characterized in several eukaryotes in recent studies and was involved in regulating gene expression (Liang et al., 2018; Xiao et al., 2018; Liu et al., 2019; Luan et al., 2019). The aberrational transcription of non-coding gene, as an important epigenetic control element, could also exert a great impact on important biological processes (Ling et al., 2013; Luo et al., 2017; Gomez-Orte et al., 2019). In this research, we first analyzed the 6mA modification distribution in ncRNA and compared it with protein-coding genes in model species included *H. sapiens*, *D. melanogaster*, *C. elegans*, and *A. thaliana*. The mitochondria DNA had a higher 6mA density than autosomal chromosomes in all four species (Figure 1 and Supplementary Figure S1) which was consist with previous study (Xiao et al., 2018). In addition, the consensus motif of 6mA methylation was highly conserved in four multicellular eukaryotic organisms (Figure 2). We further found that 6mA-methylated genes expressed significantly different from unmethylated genes except for *C. elegans*, which implied that the 6mA might be pertaining to the gene transcription (Figure 4). Importantly, the ncRNA gene was less easily to be methylated than protein-coding gene (Figure 3A). Furthermore, the 6mA density of methylated tRNA, snRNA, and miRNA genes were significantly higher than protein-coding genes (Supplementary Figure S4A). However, the 6mA density in non-coding genes was commonly higher than protein-coding genes.

The 6mA density of gDNA in *A. thaliana* (0.099%), *C. elegans* (0.027%), *D. melanogaster* (0.023%), and *H. sapiens* (0.083%) (Table 1) was different from previous studies *A. thaliana* (∼0.048%) (Liang et al., 2018), *C. elegans* (∼0.7%) (Greer et al., 2015), *D. melanogaster* (∼0.07%) (Zhang et al., 2015), and *H. sapiens* (∼0.051%) (Xiao et al., 2018), which indicating that 6mA was dynamic in various tissues and development stages. Although 6mA abundance varied in various species genomes, the consensus motifs of short nucleotides at 6mA location were highly conserved (Figure 2). The four species shared conserved sequence of short nucleotides, such as AGG motif in 6mA location, which suggested that the 6mA DNA methyltransferases in multicellular eukaryotes might have conserved catalytic functional domain.

In human, the 6mA density of lncRNA was significantly lower than protein-coding gene which was inconsistently observed in other three species (Supplementary Figure S3). We further investigated the FPKM values of 6mA-methylated lncRNA and protein-coding genes and compared them with -unmethylated genes through analyzing the RNA-seq datasets from the same tissues in four species. For lncRNA, the expression of 6mA-methylated lncRNA genes was distinctly lower than -unmethylated genes in *A. thaliana*, *D. melanogaster*, and *H. sapiens*, but the trend was not observed in *C. elegans* (Figure 4B and Supplementary Data Sheet S4). Considering the cases of *C. elegans* was probably because of missing lncRNA annotations, and the 6mA-methylated lncRNA genes may correlate with gene repression. For protein-coding genes, the methylated genes expressed significantly higher than -unmethylated genes in human (Figure 4B), but the contrast trend was observed in *A. thaliana* and *D. melanogaster*. The correlation between 6mA and protein-coding gene expression in different individuals of the same organism and various organisms may be dynamically regulated in different developmental conditions (Liang et al., 2018).

The short protein-coding genes and lncRNA genes tended to contain higher 6mA density in four species. To explore the relationship between gene length and transcription regulation, we performed correlation test between gene length and gene expression in lncRNA and protein-coding genes. For lncRNA, there was no significant correlation between gene length and gene expression. For protein-coding genes, we found a weak negative association between genes length and gene expression in four species (*A. thaliana*: *r* = −0.016, *p* = 0.007, *C. elegans*: *r* = −0.047, *p* = 3.159e−11, *D. melanogaster*: *r* = −0.022, *p* = 0.009, and *H. sapiens*: *r* = −0.040, *p* = 1.356e−09), which revealed gene length could affect the gene expression level (Chiaromonte et al., 2003). In addition, the expression of both lncRNA and protein-coding genes may be affected by multiple factors such as various types of DNA methylation. Whether the 6mA modification plays a different role in lncRNA and protein-coding genes in various organisms will be an interesting topic for further investigation.

Due to the number of 6mA-methylated tRNA, rRNA, miRNA, and snRNA genes were small and lncRNA was the widely studied ncRNA, we detailed the 6mA distribution of lncRNA and compared it with protein-coding genes in this study. Although there is a lack of GO terms for lncRNA, we analyzed the potential function of lncRNA in *H. sapiens* by using the lncRNA target genes database lncRNA2target^2^. Then we further performed GO enrichment analysis of 6mA-methylated lncRNA target genes. The statistical *P*-values from Fisher’s exact test were adjusted by the Benjamini and Hochberg’s approach and the adjusted *P*-value (FDR) < 0.05 was considered statistically significant. The result of GO analysis revealed that the 6mA-methylated lncRNA target genes played a role in regulating of vasculature development and angiogenesis (Supplementary Figure S5 and Supplementary Data Sheet S5). Considering the human datasets were from the blood, the result indirectly implied that the 6mA-methylated lncRNA genes had a biological function in specific tissue. The potential function of 6mA modifications in lncRNA need to be further investigated.

## Data Availability Statement

Publicly available datasets were analyzed in this study. These data can be found in the NCBI database: SRP073602, SRP159040, SRP186435, SRP126018, SRP171981, and SRP068953.

## Author Contributions

S-QX and JC conceived the project and designed the experiments. YL, M-WL, and J-FX collected datasets and performed the bioinformatics analysis. YL plotted figures. YL, X-MZ, and S-QX wrote the manuscript. All authors read and approved the final manuscript.

## Conflict of Interest

The authors declare that the research was conducted in the absence of any commercial or financial relationships that could be construed as a potential conflict of interest.

## Footnotes

1 https://www.pacb.com/products-and-services/analytical-software/epigenetics/

2 http://123.59.132.21/lncrna2target
