# Corrigendum: Distribution Patterns of DNA N6-Methyladenosine Modification in Non-coding RNA Genes

**DOI:** 10.3389/fgene.2020.00904

**Published:** 2020-08-25

**Authors:** Yu Li, Xiao-Ming Zhang, Mei-Wei Luan, Jian-Feng Xing, Jianguo Chen, Shang-Qian Xie

**Affiliations:** ^1^Key Laboratory of Genetics and Germplasm Innovation of Tropical Special Forest Trees and Ornamental Plants (Ministry of Education), Hainan Key Laboratory for Biology of Tropical Ornamental Plant Germplasm, College of Forestry, Hainan University, Haikou, China; ^2^College of Grassland, Resources and Environment, Inner Mongolia Agricultural University, Huhhot, China; ^3^School of Life Sciences, Hubei University, Wuhan, China

**Keywords:** non-coding RNAs, model species, DNA methylation, gene expression, 6mA modification

In the original article, there was an error in the abstract. The sentence “Especially, the 6mA-methylated lncRNA genes were expressed significant lower than these genes without methylation in *A. thaliana* (*p* = 3.295e-4), *D. melanogaster* (*p* = 3.439e-11), and *H. sapiens* (*p* = 9.087e-3) all four species.” should be revised to “Especially, the 6mA-methylated lncRNA genes were expressed significant lower than genes without methylation in *A. thaliana* (*p* = 3.295e-4), *D. melanogaster* (*p* = 3.439e-11), and *H. sapiens* (*p* = 9.087e-3).” A correction has been made in the Abstract.

N6-methyladenosine (6mA) DNA modification played an important role in epigenetic regulation of gene expression. And the aberrational expression of non-coding genes, as important regular elements of gene expression, was related to many diseases. However, the distribution and potential functions of 6mA modification in non-coding RNA (ncRNA) genes are still unknown. In this study, we analyzed the 6mA distribution of ncRNA genes and compared them with protein-coding genes in four species (*Arabidopsis thaliana, Caenorhabditis elegans, Drosophila melanogaster*, and *Homo sapiens*) using single-molecule real-time (SMRT) sequencing data. The results indicated that the consensus motifs of short nucleotides at 6mA location were highly conserved in four species, and the non-coding gene was less likely to be methylated compared with protein-coding gene. Especially, the 6mA-methylated lncRNA genes were expressed significant lower than genes without methylation in *A. thaliana* (*p* = 3.295e-4), *D. melanogaster* (*p* = 3.439e-11), and *H. sapiens* (*p* = 9.087e-3). The detection and distribution profiling of 6mA modification in ncRNA regions from four species reveal that 6mA modifications may have effects on their expression level.

The authors apologize for this error and state that this does not change the scientific conclusions of the article in any way. The original article has been updated.

