# Supplementary material for: Distribution Patterns of DNA N6-Methyladenosine Modification in Non-coding RNA Genes
Source: Front Genet. 2020 Mar 24;11:268. doi: 10.3389/fgene.2020.00268 (PMC7105833; doi:10.3389/fgene.2020.00268)
Supplement: Supplementary file 1 [file Presentation_1.pdf]

Table S1. Datasets collected in this study

Species	SRA accession	Type	Tissues	development stage	# of Bases	Reference link
A. thaliana	SRP073602	DNA	leaves	mature	109.5G	https://www.ncbi.nlm.nih.gov/genome/?term=Arabidopsis+thaliana
	SRP159040	RNA	leaves	65 days	283M	
C. elegans	SRP186435	DNA	whole worm	mixed-stage	62.7 G	https://www.ncbi.nlm.nih.gov/genome/?term=Caenorhabditis+elegans
	SRP186435	RNA	whole worm	mixed-stage	3.1G	
D. melanogaster	SRP126018	DNA	Body	Adult	56.7G	https://www.ncbi.nlm.nih.gov/genome/?term=Drosophila+melanogaster
	SRP171981	RNA	Body	Adult	6.1G	
H. sapiens	SRP068953	DNA	blood	40	2085.6G	https://www.ncbi.nlm.nih.gov/genome/?term=Homo+sapiens
	SRP068953	RNA	blood	40	38.8G	

Table S2. The threshold of gene length for grouping lncRNA and protein-coding genes.

Species	lncRNA genes length (x100 bp)			Protein-coding genes length (x100 bp)			
	Group1	Group2	Group3	Group1	Group2	Group3	Group4
A. thaliana	<5	5~10	>10	<10	10~20	20~30	>30
C. elegans	<10	10~20	>20	<10	10~20	20~30	>30
D. melanogaster	<10	10~20	>20	<20	20~40	40~60	>60
H. sapiens	<100	100~200	>200	<150	150~300	300~450	>450

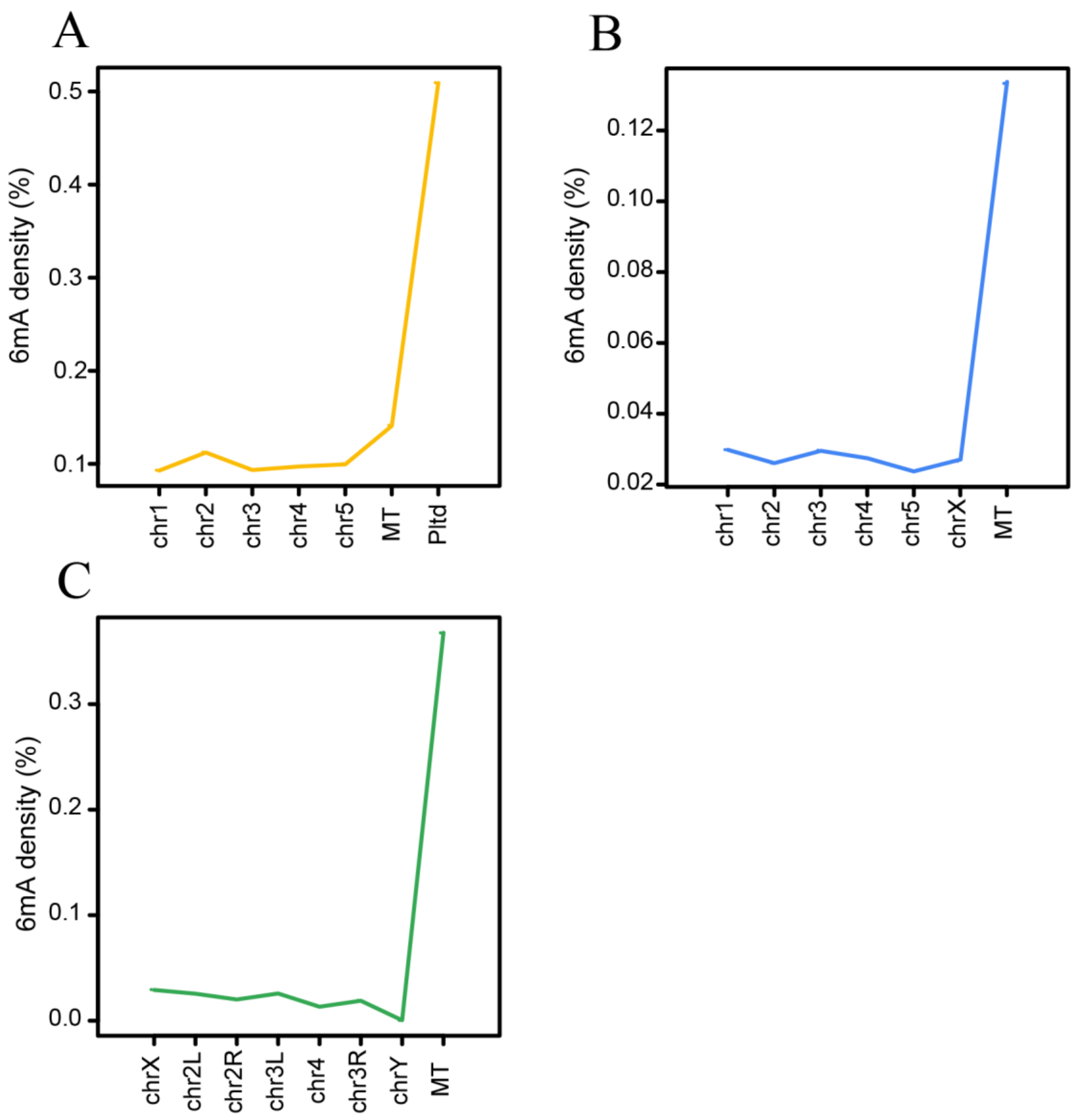

Figure S1. Distribution of N6-methyladenosine modification (6mA) in three species genomic DNA. (A) Line diagram of 6mA modification density in *A. thaliana* genome. (B) Line diagram of 6mA modification density in *C. elegans* genome. (C) Line diagram of 6mA modification density in *D. melanogaster* genome.

A

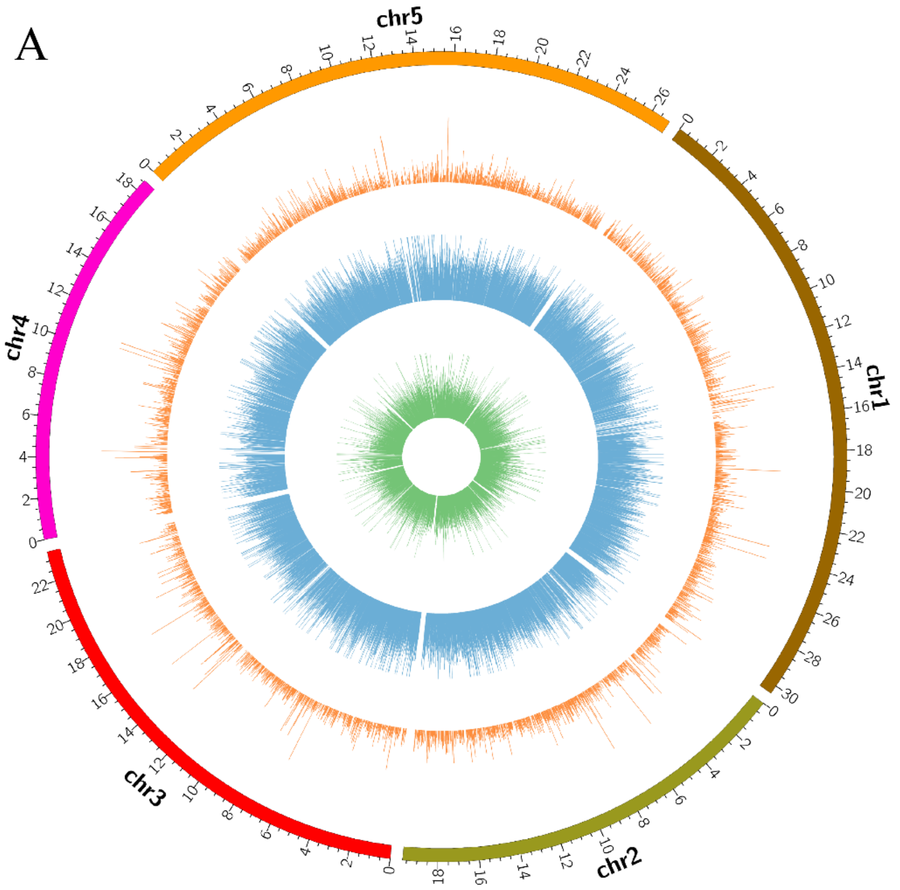

B

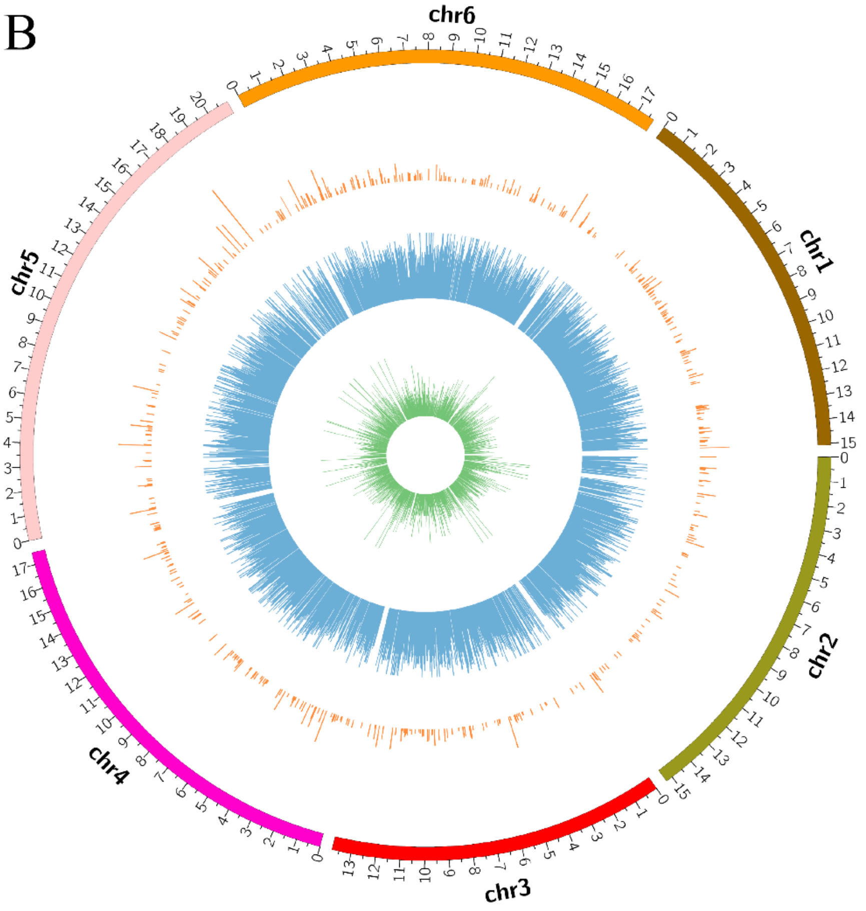

C

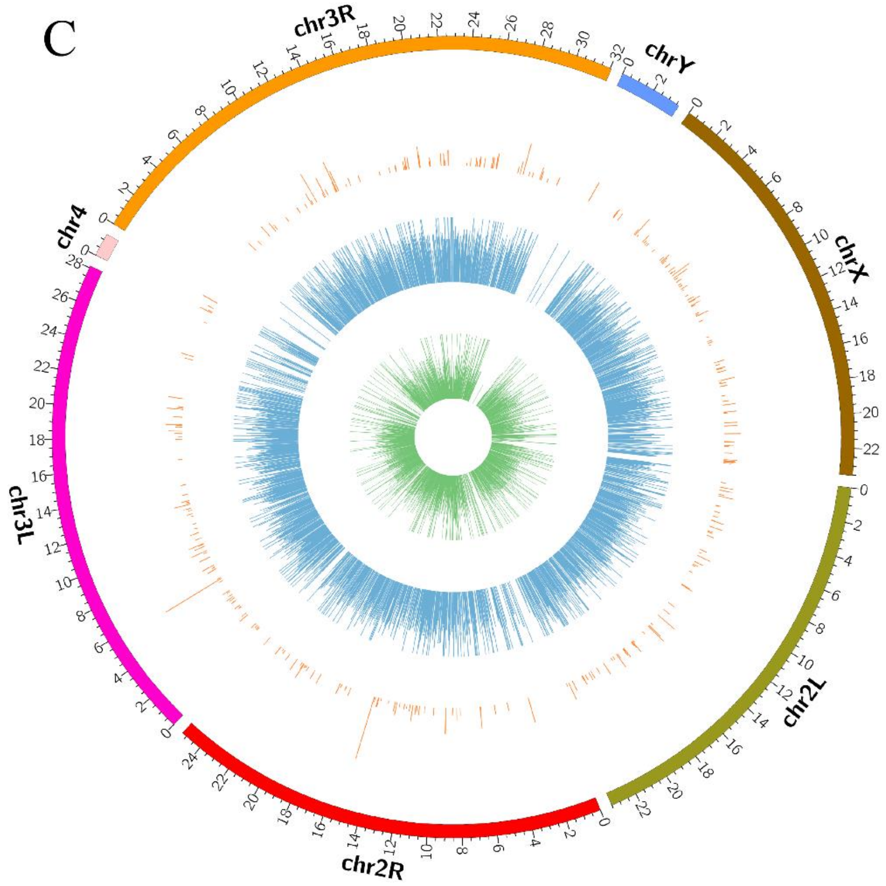

Figure S2. Distribution of N6-methyladenosine modification (6mA) in three species genomic DNA. (A) Circos plots of 6mA DNA methylation profiles of *A. thaliana.* (B) Circos plots of 6mA DNA methylation profiles of *C. elegans.* (C) Circos plots of 6mA DNA methylation profiles of *D. melanogaster*. (Green ring: lowly methylated (0–30%) 6mA. Blue ring: moderately methylated (30%–70%) 6mA. Orange ring: highly methylated (70%–100%) 6mA.)

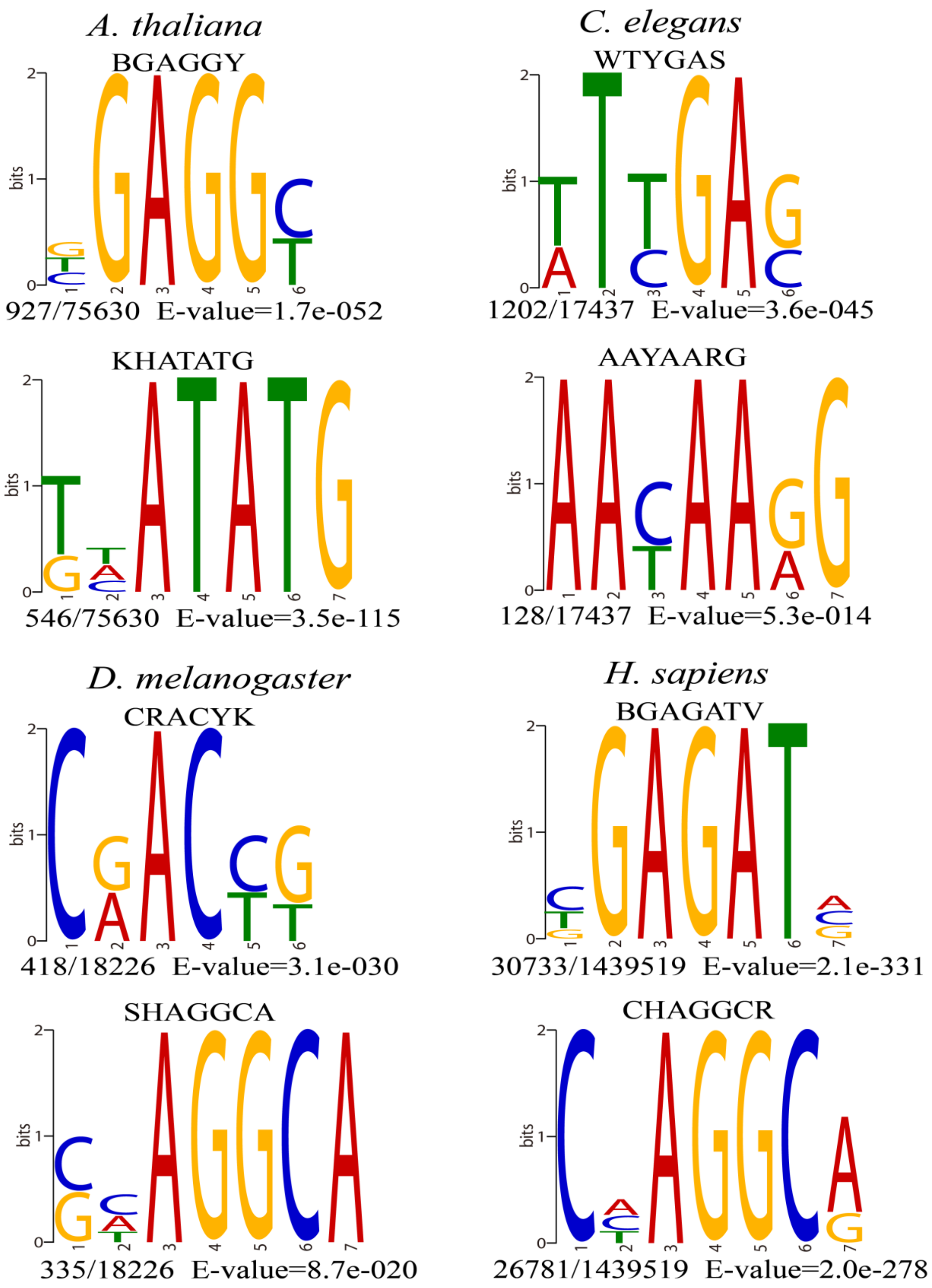

Figure S3. The identified consensus motifs containing 6mA sites in four species. (The number of occurrences of each motif relative to the total number of 6mA-containing motifs and the corresponding E-value generated by DREME are shown under the sequence logo.)

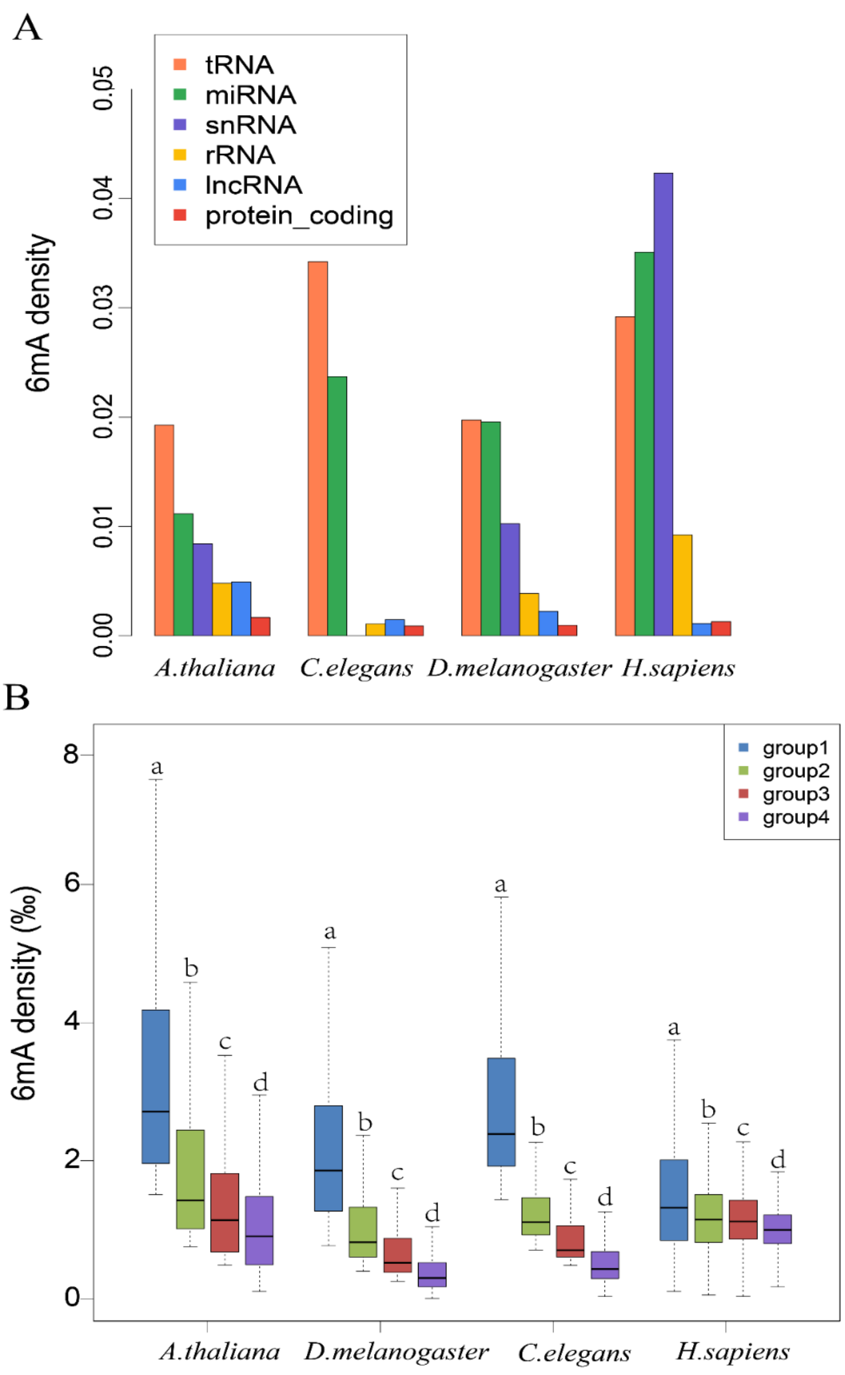

Figure S4. Distribution pattern of 6mA modification in non-coding genes in four species. (A) The percentages of genes containing 6mA modification in different gene categories were shown in histogram. (B) The 6mA density of different protein-coding gene length groups was shown in boxplot. The different letters shown above the box meant the significant difference in statistical analysis.

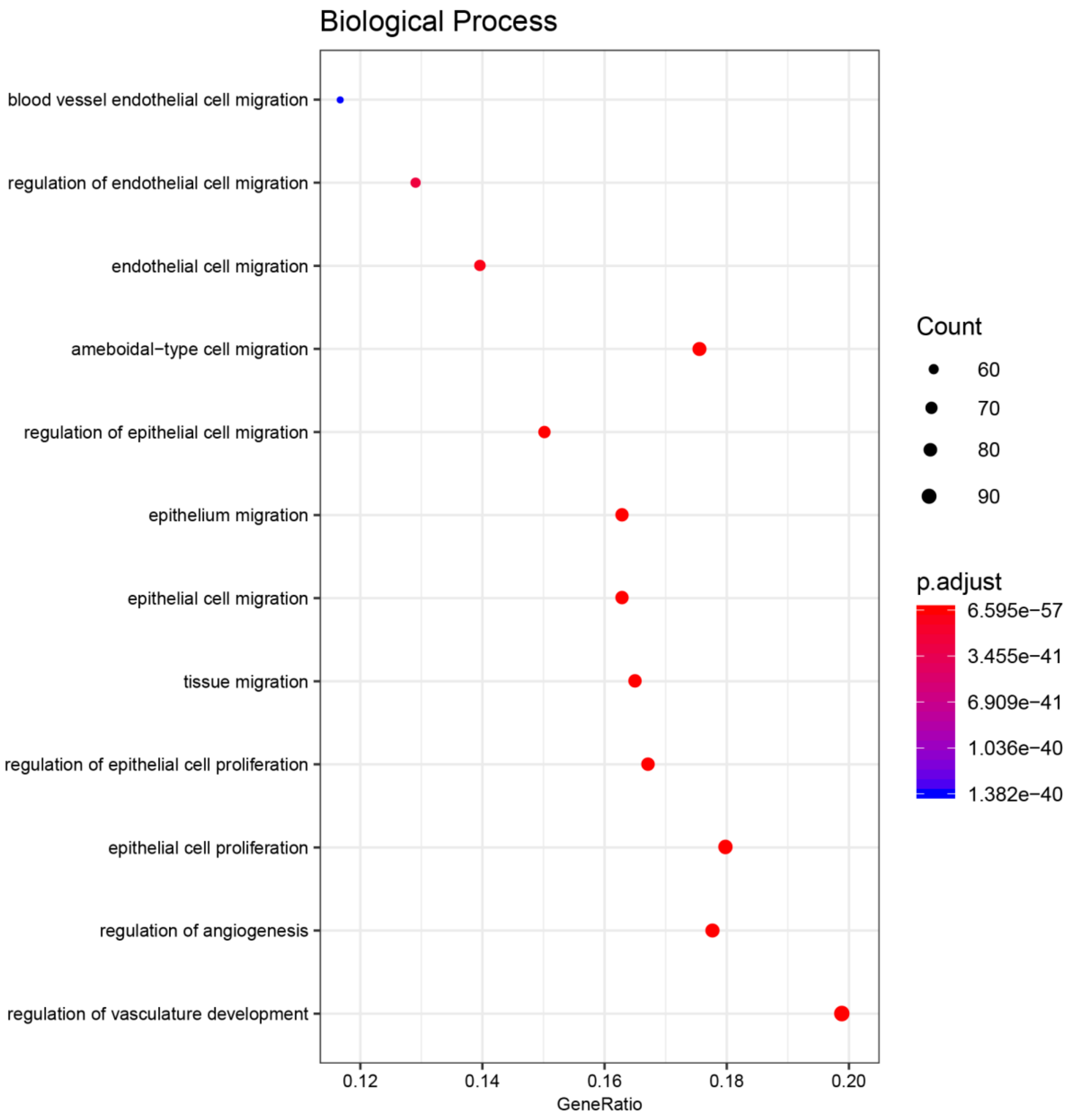

Figure S5. GO enrichment category of 6mA-methylated lncRNA target genes in biological process in *H. sapiens*. (P-values were adjusted by the Benjamini and Hochberg's approach, the GO category was listed with the adjusted P-value (FDR) <0.05.)
